# Age-related changes in expression and signaling of TAM receptor inflammatory regulators in monocytes

**DOI:** 10.18632/oncotarget.23851

**Published:** 2018-01-03

**Authors:** Xiaomei Wang, Anna Malawista, Feng Qian, Christine Ramsey, Heather G. Allore, Ruth R. Montgomery

**Affiliations:** ^1^ Department of Internal Medicine, Yale University School of Medicine, New Haven, Connecticut; ^2^ Yale Center for Medical Informatics, Yale University School of Medicine, New Haven, Connecticut; ^3^ Human Translational Immunology, Yale University School of Medicine, New Haven, Connecticut; ^4^ State Key Laboratory of Genetic Engineering and Ministry of Education Key Laboratory of Contemporary Anthropology, School of Life Sciences, Fudan University, Shanghai, China

**Keywords:** 5 immunosenescence, monocyte, age-related immune dysregulation, TAM receptors, protein S, Gerotarget

## Abstract

The multifactorial immune deterioration in aging--termed “inflamm-aging”--is comprised of a state of low-grade, chronic inflammation and complex dysregulation of responses to immune stimulation. The TAM family (Tyro 3, Axl, and Mer) of receptor tyrosine kinases are negative regulators of Toll like receptor-mediated immune responses that broadly inhibit cytokine receptor cascades to inhibit inflammation. Here we demonstrate elevated expression of TAM receptors in monocytes of older adults, and an age-dependent difference in signaling mediator AKT resulting in dysregulated responses to signaling though Mer. Our results may be especially significant in tissue, where levels of Mer are highest, and may present avenues for modulation of chronic tissue inflammation noted in aging.

## INTRODUCTION

Good health in aging is compromised by a progressive decline in immune function (immunosenescence) which results in more severe consequences of viral and bacterial infections and decreased response to vaccines [1, 2]. Numerous studies in older adults have revealed a profound impact on the phenotype and functions of immune cells in aging, which in the innate immune system, lead to dysregulation of responses with inappropriate elevations and decreases in immune responses [3–5]. This multifactorial immune deterioration--termed “inflamm-aging”--is comprised of a state of low-grade, chronic inflammation characterized by broad dysregulation of cellular stress pathways, oxidative stress, mitochondrial dynamics and autophagy, chronic viral infections, obesity, DNA damage response, and impaired clearance of damaged cells [6–8].

Innate immune cells sense diverse microorganisms through the evolutionarily conserved signaling pattern recognition receptors, Toll-like receptors (TLRs), that recognize highly conserved components of microbial pathogens and mediate the earliest host response to infection [9, 10]. Additional recognition mechanisms include the cytoplasmic retinoic acid-inducible gene I (RIG-I)-like receptors (RLRs), cytosolic DNA sensors, and nucleotide-binding oligomerization domain (NOD)-like receptors (NLRs) that trigger inflammasome activation [11, 12]. With age, expression and function of TLRs, RIG-I, and NLRs declines in several cell lineages including monocytes, dendritic cells (DCs) and polymorphonuclear leukocytes (PMN) [4, 13–20]. Age-associated decreases in pattern recognition receptor expression and downstream signaling cascades result in reduced cytokine production, particularly those related to interferon-dependent signaling, and are associated with lower vaccine responsiveness [1, 4, 21–24].

The powerful TLR activation pathways are regulated to limit inflammation. Indeed, successful immune responses represent a balance of pro- and anti-inflammatory processes. Recent studies have identified the TAM family (Tyro 3, Axl, and Mer) of receptor tyrosine kinases as negative regulators of TLR-mediated immune responses that broadly inhibit both TLR and TLR-induced cytokine receptor cascades to limit inflammation [25–29]. The importance of TAMs in immune activation is illustrated by the observation that reduced levels of TAMs in humans and in a TAM-deficient mouse model are associated with susceptibility to autoimmune disease and higher or chronic inflammation [27, 30, 31]. Further, TAMs play a key role in mediating autophagy [26, 27, 32], and the reduced efficiency of autophagy in aging has been shown to contribute to accumulation of damaged proteins in cells [33].

In previous studies of aging, we have shown dysregulation of immune responses with a paradoxical increase in levels of pro-inflammatory cytokines such as IL-6 and TNF-α and a decrease in anti-viral interferon responses [1, 3, 5, 34]. In examining the role of negative regulation in age-related immune deficiencies, we showed higher expression of Axl in monocyte-derived DCs from older individuals after infection with West Nile virus [21]. These data suggested that enhanced negative signaling in aging may contribute to a more severe outcome in viral infection. The current studies aim towards a fuller molecular understanding of the effects of aging on expression and function of TAMs and may provide insights to immune mechanisms involved in chronic inflammation that contribute to multiple immune dysfunctions in aging.

## RESULTS

### TAM receptor expression is elevated in aging

**Figure 1:**
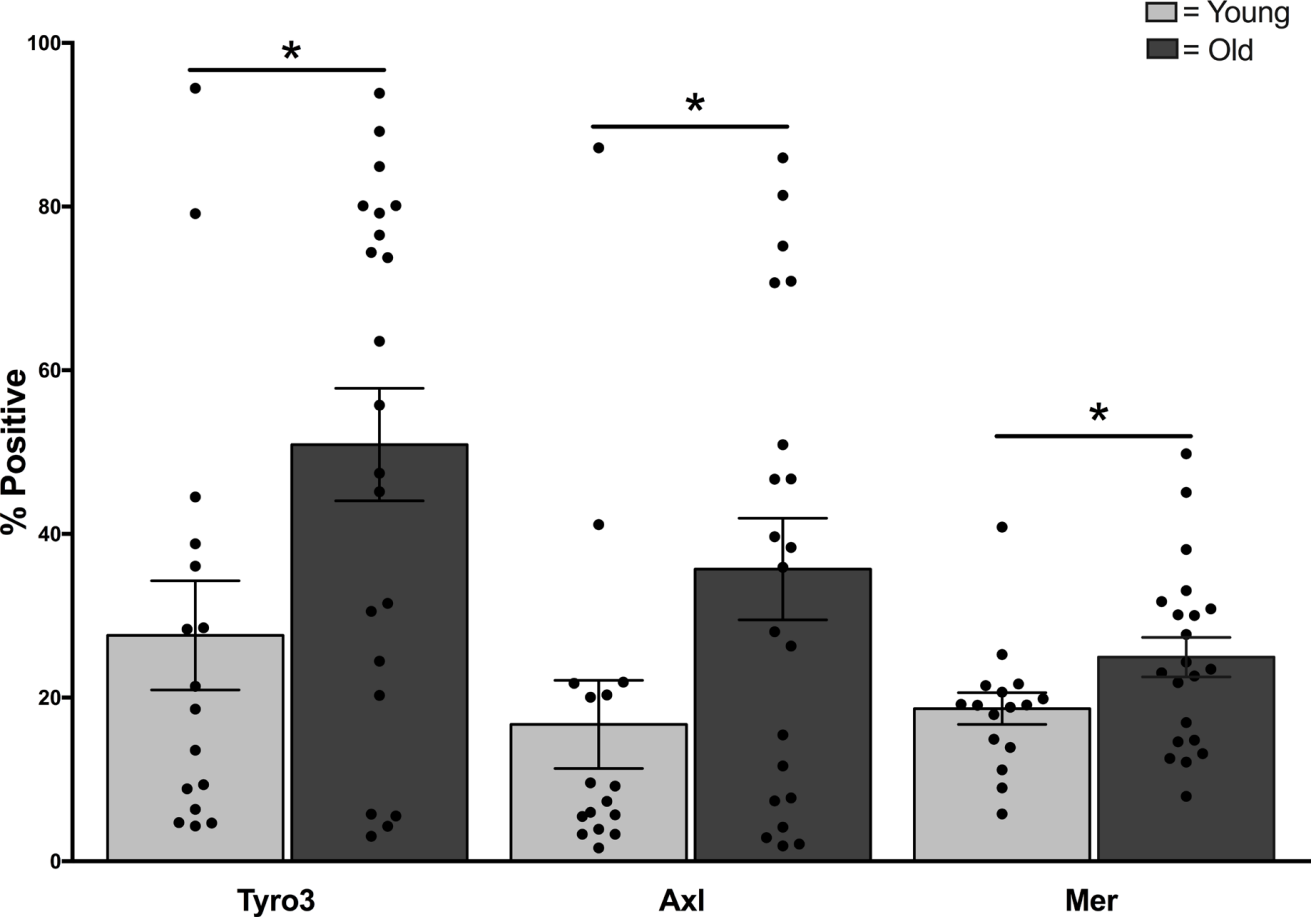
Elevated expression of TAM receptors in monocytes of older adults PBMCs from younger and older adults were labeled for TAM receptors Tyro3, Axl, and Mer and expression was quantified by LSR-II flow cytometry as described [39]. Data shown is percent expression of TAM receptor in CD14^+^, CD11c^+^ monocytes, *n* = 16 young, *N* = 21 old, ^*^ indicates *p* < 0.02 using multivariate Generalized Linear Models.

To identify effects of aging on TAM receptors, we recruited younger (age 21–30 years, *N* = 40) and older (age 65–89, *N* = 50) adults and assessed TAM expression and function. Participants were healthy at the time of sampling but varied significantly by age group as expected for number chronic conditions, and prescription and over the counter medications (Table 1). We quantified surface expression of TAM receptors as a measure of potential functional TAM activity between age groups. Flow cytometry of CD14^+^, CD11c^+^ monocytes revealed highly variable expression among healthy donors of both age groups, particularly for Tyro3 and Axl, with some donors expressing almost no Axl or Tyro3 at all (Figure 1 and Supplementary Figure 1). In contrast, Mer was expressed in all donors and was less variable. The surface expression (% positive) of all three TAM receptors was elevated in monocytes of older donors (Tyro3: Young 26.8 + 7.7 vs Old 54.5 + 7.8, *p* = 0.02; Axl: Young 16.3 + 6.5 vs Old 40.4 + 6.7, *p* = 0.02; Mer: Young 18.6 + 2.4 vs Old 27.6 + 2.4; *p* = 0.03). The mean fluorescence intensity (MFI) was not different between age groups.

**Table 1:** Human participants

Patient Characteristics	Young	Older	
(*N* = 90)	(*N* = 40)	(*N* = 50)	*P*-value
Age (years) range (22–89), mean (SD)	25.8 (2.2)	73.6 (6.4)	< 0.001
Female, *n* (%)	27 (67.5)	23 (46.0)	0.055
White Race, *n* (%)	25 (62.5)	46 (92.0)	0.001
Number of Comorbid Conditions, mean (SD)	0.9 (0.9)	2.8 (1.8)	< 0.001
Myocardial Infarction, *n* (%)	0 (0.0)	3 (6.0)	0.249
Congestive Heart Failure, *n* (%)	0 (0.0)	1 (2.0)	1.000
Coronary Artery Disease, *n* (%)	0 (0.0)	9 ( 18.0)	0.004
Cardiac Arrhythmia, *n* (%)	0 (0.0)	4 ( 8.0)	0.124
Hypertension, *n* (%)	2 (5.0)	31 ( 62.0)	< 0.001
Peripheral Vascular Disease, *n* (%)	0 (0.0)	2 ( 4.0)	0.498
Chronic Obstructive Pulmonary Disease, *n* (%)	0 (0.0)	1 (2.0)	1.000
Peptic Ulcer Disease, *n* (%)	1 (2.5)	2 (4.0)	1.000
Number of Prescription Medications, Age mean (SD)	0.7 (0.8)	3.5 ( 2.7)	< 0.001
Statin Medications, *n* (%)	0 (0.0)	28 ( 56.0)	< 0.001
Number of OTC Medications, mean (SD)	1.7 (1.2)	2.9 ( 1.3)	< 0.001
Aspirin, *n* (%)	0 ( 0.0)	32 (64.0)	< 0.001

^a^Probability for Student *t*-test for continuous measures, Fisher Exact test for categorical measures.

### Effects of aging on activation of Mer

Upon activation, TAM receptors initiate engulfment of apoptotic cells through Ca^+2^-dependent, phosphatidyl serine (PtdSer) mediated binding of gamma-carboxyglutamic (Gla)-domains of ligands protein S, a plasma glycoprotein usually associated with a role in the coagulation cascade, or the structurally related protein growth arrest-specific gene 6 (Gas6) [26, 35]. The three TAM receptors Tyro3, Axl, and Mer show distinct features and ligands [35, 36]. To assess the functional consequence of the age-related elevation in TAM expression, we focused on Mer, which is expressed by all donors, and highly expressed in macrophages suggesting a key role in tissue particularly relevant for apoptotic cell clearance [37–39]. Adherence-purified monocytes were incubated overnight in the absence of serum to decrease the contribution of Mer ligand Protein S from serum in culture medium. Cells were treated with PtdSer liposomes containing Protein S to engage Mer receptors [35, 36, 40]. We detected a transient increase in phosphorylated forms of Mer at 10 min which declined by 30 min and was not different between age groups (Supplementary Figure 2A). This is consistent with a Protein S-dependent phosphorylation of Mer following treatment *in vitro* with gas6 which has been shown previously to be maximal at 5 min [41]. Treatment with liposomes or Protein S alone did not lead to stimulation of Mer pathways (data not shown).

**Figure 2:**
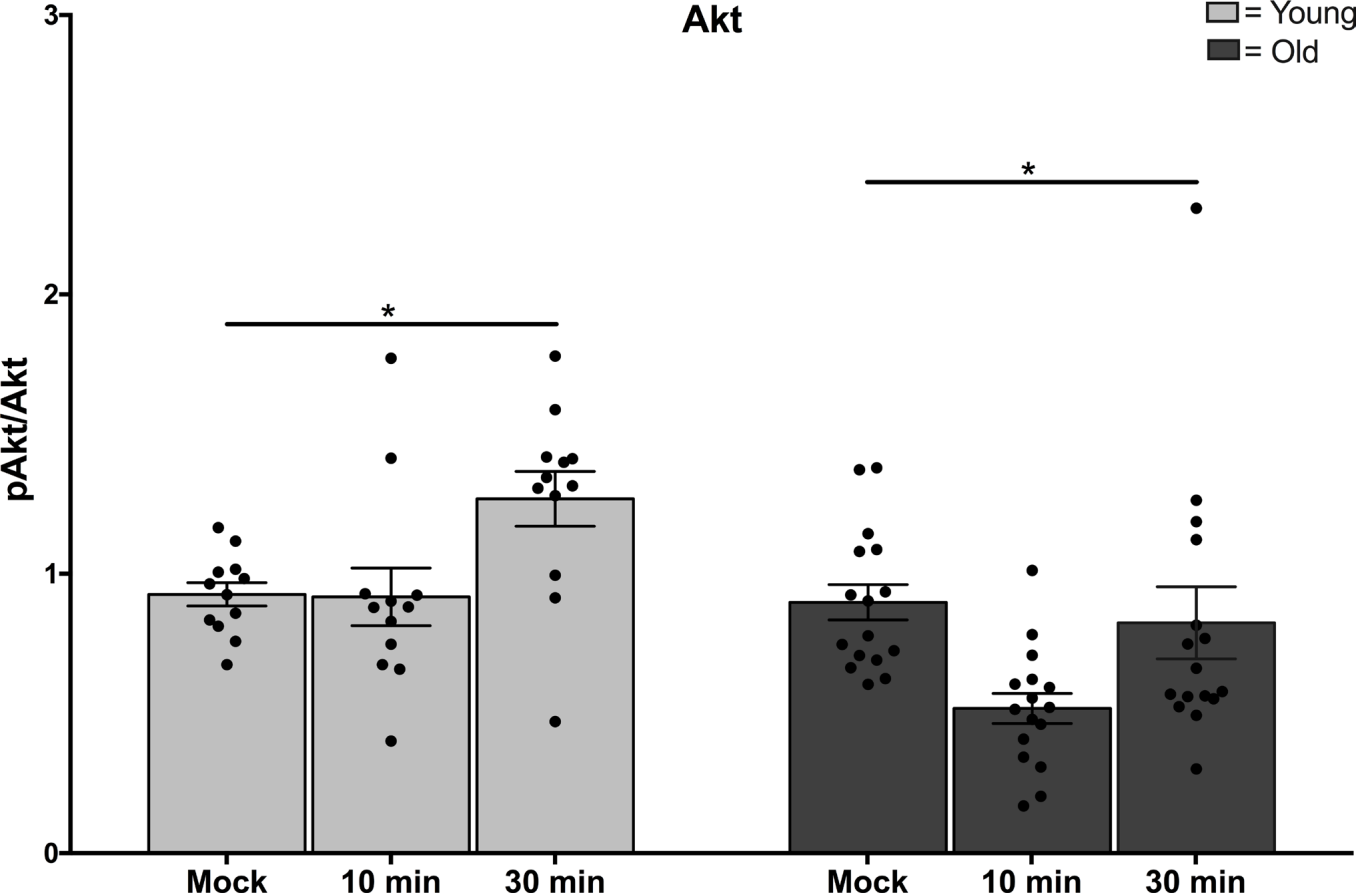
Functional changes in Mer signaling in aging Monocytes from younger and older adults were incubated overnight in the absence of serum before treatment with 0.5 mM phosphatidylserine liposomes containing 100 nM Protein S. Cells were harvested at the time points indicated and lysates were processed for Immunoblot. Data shown is densitometry for *N* = 12 young, *N* = 16 old, ^*^ indicates *p* = 0.003 in multivariate Generalized Linear Models.

Notably, treatment with PtdSer-Protein S liposomes led to increased phosphorylation of the serine/threonine kinase AKT in monocytes from younger adults at 30 min (Figure 2, *p* = 0.005), but levels of phospho-AKT in older adults were not increased (Figure 2, *p* = 0.47). This age-dependent difference in signaling mediator AKT is significant for both the higher levels in younger adults and also the change over time following stimulation (Figure 2, *p* = 0.003). In the same samples, reduction in levels of signaling mediators pSTAT1 and p38 was detected at 10 and 30 min following treatment with PtdSer-Protein S liposomes, however the decreases noted were not different between age groups (Supplementary Figure 2B, 2C).

**Figure 3:**
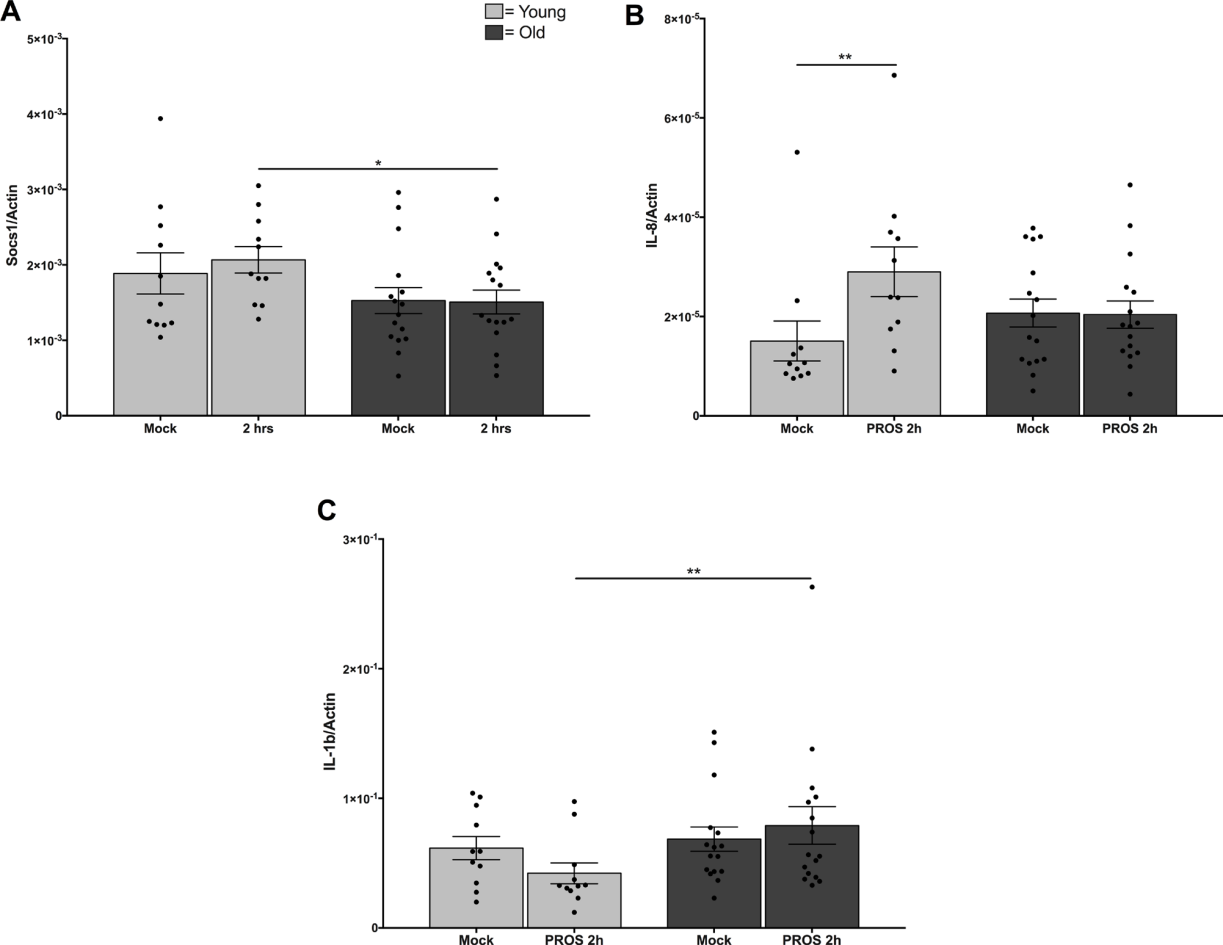
Reduced responses to Mer signaling in monocytes from older adults Monocytes from younger and older adults were incubated overnight in the absence of serum before treatment with 0.5 mM phosphatidylserine liposomes containing 100 nM Protein S. RNA was extracted for Q-PCR. Data shown is (**A**) Soc1, (**B**) IL-8, and (**C**) Il-1β expressed as a ratio to cellular b-actin from cells untreated or stimulated for 2 hours from *N* = 12 young, *N* = 16 old, ^*^ indicates *p* = 0.03 using Type 3 Test of Fixed Effects.

Phosphorylation of AKT in monocytes would be expected to lead to elevated transcription of downstream mediators of inflammatory responses. In correlation with the levels of phospho-AKT, treatment of monocytes from younger adults with PtdSer-Protein S liposomes led to increased levels of SOC-1 and IL-8, with a significant change from baseline to 2 hours in younger adults which was not seen in monocytes from older adults (Figure 3A, 3B, *p* = 0.03). Further, we noted age-related differences in IL-1β responses to PtdSer-Protein S liposomes. Notably, the decrease in levels in younger adults from baseline over 2 hours of treatment and increase in levels in the older adults over the 2 hours of treatment result in a significant difference between younger and older adults (Figure 3C, *p* = 0.03). In addition, for a subset of subjects with data collected at 8 hours, we noted extension of the same trajectory (data not shown). Age-related differences were not noted in levels of IFNβ and TNF (not shown), suggesting the kinetics of activation of those targets were not captured by our study.

## DISCUSSION

**Figure 4:**
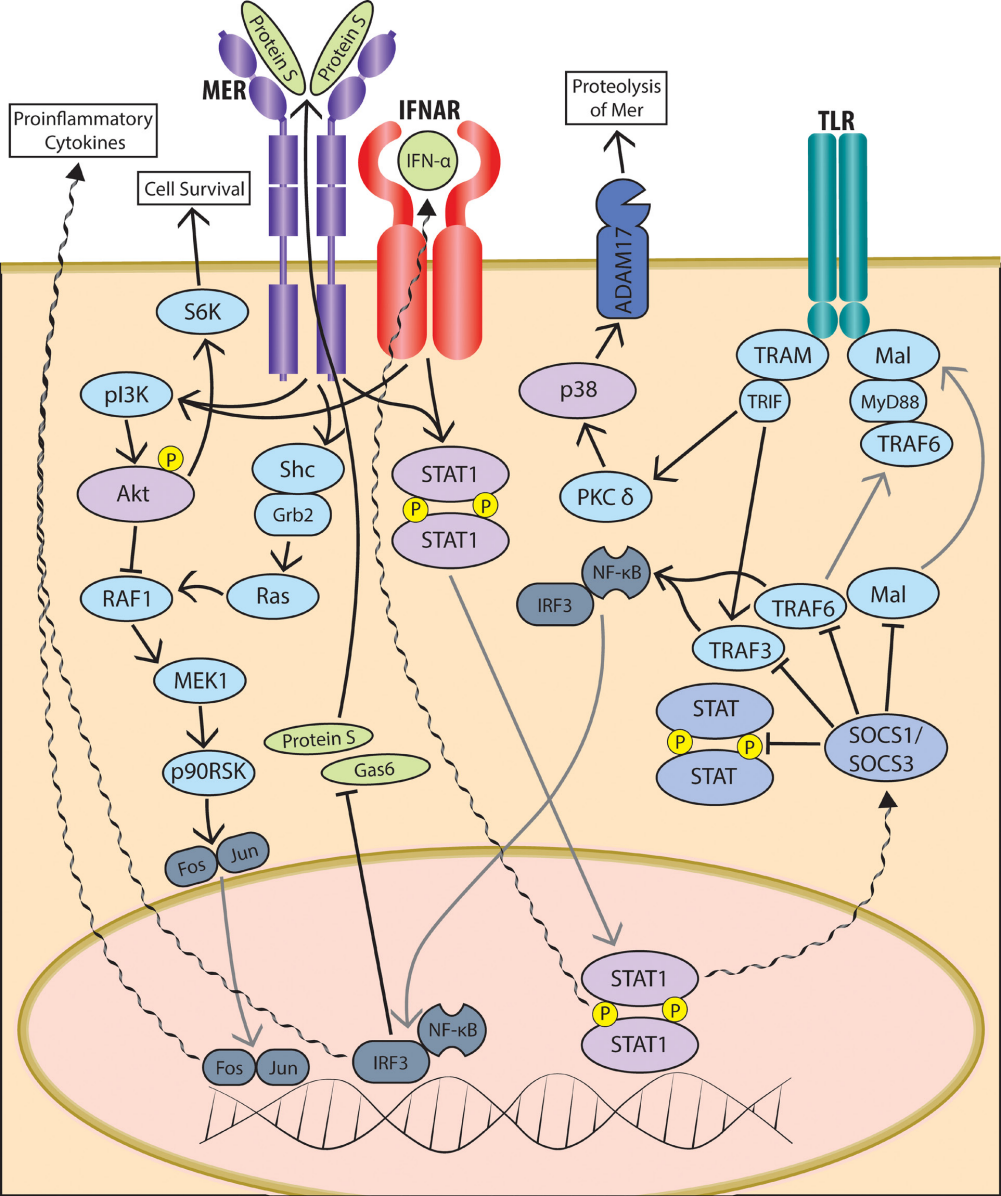
Schematic of TAM pathway signaling in innate immune cells Schematic representation of some of the most relevant signalling intermediates in the intracellular signaling cascades triggered in response to ligation of TAM family member Mer by its ligand Protein S.

The elevated expression of TAM receptors in monocytes from older adults has important implications for dysregulation of immune responses in aging--in particular as the Mer pathway is critical for clearance of apoptotic cells that contribute to inflammation in aging [37, 38]. We have previously used paired samples of monocytes and mature macrophages from healthy adults to show significant upregulation of Mer along the maturation of monocytes to macrophages, suggesting that levels of Mer are highest in tissue, which is relevant for its role in clearance of apoptotic cells [39]. Here we show age-related dysregulated activation of the Mer pathway following binding of Protein S, leading to impaired signaling through AKT in monocytes of older adults. Our findings suggest a possible role for phosphatidylinositol-3 kinase (PI3K) in this age-related deficiency in phosphorylation of AKT. This is consistent both with the established role for PI3K/AKT pathway in Mer activation [42], previous reports of dysregulation of PI3K in aging [43–45], as well as the absence of age-associated changes in Mer stimulated signaling through STAT-1 in the current study (Figure 4).

TAM pathways are important in a range of biological functions. Unexpectedly, TAMs have been shown to provide an immune evasion mechanism for certain enveloped viruses through “apoptotic mimicry”, in which they enhance infectivity by bridging virion phosphatidyl serine via gas6 binding to increase uptake through TAM receptors [46–48]. Thus, the elevated levels of TAMs in innate immune cells in aging may contribute to susceptibility to certain viral infections [48]. This may be important in response to West Nile virus, for which aging is a significant risk factor [34], although recent studies with the closely related Zika virus suggest this may be specific to particular viruses [49]. Studies of immune regulation in tumor progression have highlighted a role for TAMs, with inhibition of TAM tyrosine kinase pathways being actively investigated as a novel cancer therapeutic [50–52]. In addition, advances in our understanding of host genetic variation such as polymorphisms in Mer, which are associated with susceptibility to autoimmune disease, or genes related to aging [53–57], may offer insight into mechanisms to regulate TAM pathways. Taken together, improved understanding of TAM pathways may reveal avenues for modulation of chronic tissue inflammation noted in aging and may present targeted therapeutic approaches to address immune deficits in aging.

## MATERIALS AND METHODS

### Study subjects

Heparinized blood from healthy volunteers (younger 21–30, older > 65) was obtained with written informed consent under an IRB protocol approved annually by the Human Investigations Committee of Yale University. Donors had no acute illness and took no antibiotics or nonsteroidal anti-inflammatory drugs within one month of enrollment. Enrollment of these adults was conducted at flu vaccination clinics in two consecutive years (2013–2014) and demographic characteristics were collected at enrollment (Table 1). Self-reported information comprised demographic data, height, weight, medications, and comorbid conditions; immunocompromised individuals as defined previously were excluded [15]. The proportion of women or race was not statistically different between age groups. As it was not possible to assess each donor for each cell-based assay, samples were randomly chosen for experiments over > 2 years for assays under study at the time of recruitment and sample number is indicated in each figure legend.

### Flow cytometry labeling

Human peripheral blood mononuclear cells (PBMCs) were isolated from heparinized blood using Ficoll-Hypaque (GE Healthcare, NJ) as previously described [58]. For measurement of Tyro, Axl, and Mer expression, cells were labeled for 30 min at 4°C protected from light with antibodies for surface lineage markers and TAMs as follows: APC-Cy7 CD16 (BD Biosciences, CA 557758), PerCP CD14 (BD 340585), APC CD11c (BD 559877), PE anti-Axl (R & D FAB154P), anti-Mer (FAB8912P), and anti-Tyro (FAB859P) [39]. The immunostained cells were washed with BD wash buffer and fixed in 1% paraformaldehyde. Data was acquired using an LSR II instrument (BD Biosciences, CA) and analyzed using FlowJo software (Tree Star, OR) as described previously [16]. Gating strategy is shown in Supplementary Figure 1.

### Protein S signaling assays

PBMCs were plated at 2.5 × 10^6^/ml in a 12 well plate in RPMI/20% human serum/1% pen/strep, washed at 2 hr, and incubated overnight in RPMI in the absence of serum as described previously [59]. Adherent monocytes were pre-treated with medium alone or with 0.5 mM phosphatidyl serine liposomes (Avanti Polar Lipids, Inc., Alabaster, AL) complexed for 30 min under N_2_ gas with 100 nM Protein S (Haematologic Technologies, Inc., Essex Junction, VT) as described [36]. Cells were harvested at 10, 30 min for immunoblot or at 4 hr for Q-PCR.

### Quantitative PCR (qPCR) analysis

Total RNA was harvested by RNeasy mini kit (Qiagen, CA) and cDNA was synthesized using AffinityScript Multi Temperature cDNA Synthesis Kit (Stratagene, TX). Primers and probes for qPCRs were from Applied Biosystems: Tyro3, Axl, Mer, SOCS1, IL-6, IL-8, TNF, IL-1β. Amplification in duplicate was on batched samples in an iCycler (Bio-Rad, CA); values were normalized to β-actin [58].

### Immunoblot analysis

Cells were harvested using CelLytic M Cell Lysis buffer (Sigma, MO) containing protease inhibitor cocktail as described previously [59]. Immunoblots were probed with antibodies to total- and phospho-Mer, STAT-1, AKT, p38 MAPK, and β-actin, developed using Amersham ECL Reagents (GE Healthcare), and scanned using Image J software.

### Statistical analysis

Demographic characteristics of participants were compared between the age groups with Fisher exact tests for categorical factors and with a *t*-test for continuous factors. Age was sampled to differ, thus ranges are provided. We used multivariable General Linear Models to estimate the effect of age group on the cell expression of TAMs, and signaling studies were adjusted for within person correlation of test conducted at baseline, 10 and 30 minutes. Statistical tests used SAS version 9.2 (SAS Institute, Cary, NC) and were 2-tailed with *p* < 0.05 considered significant. Difference for qPCR data was determined using Mann-Whitney test with Bonferroni correction or unpaired *t*-test using Graphpad Prism (GraphPad Software, Inc.).

## SUPPLEMENTARY MATERIALS FIGURES



## Abbreviations

RPMI
PBMC
TAM
