# Supplementary material for: Age-related changes in expression and signaling of TAM receptor inflammatory regulators in monocytes
Source: Oncotarget. 2018 Jan 3;9(11):9572–80. doi: 10.18632/oncotarget.23851 (PMC5839385; doi:10.18632/oncotarget.23851)
Supplement: Supplementary file 1 [file oncotarget-09-9572-s001.pdf]

Age-related changes in expression and signaling of TAM receptor inflammatory regulators in monocytes

SUPPLEMENTARY MATERIALS

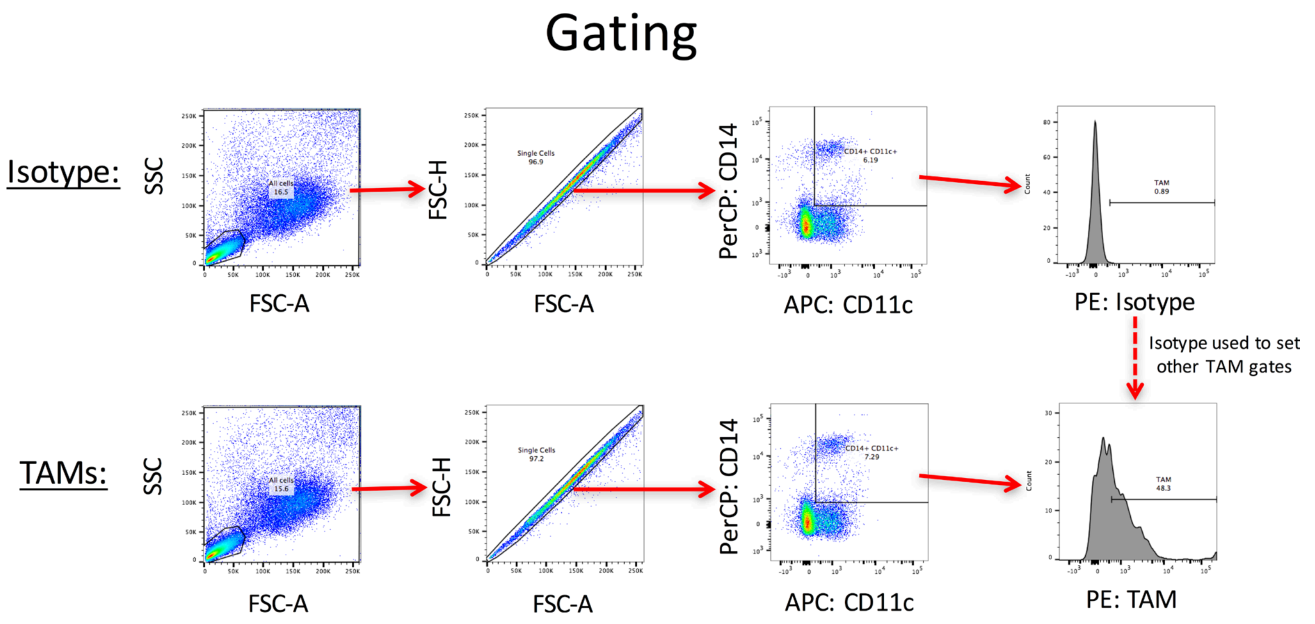

Supplementary Figure 1: Manual gating strategy to quantify TAM receptor expression on monocytes from flow cytometry dataset. PBMCs were labeled with fluorescence-conjugated antibodies against CD14, CD45, CD11c and for expression of Tyro, Axl, and Mer. PBMCs were labeled for 30 min at 4oC protected from light with antibodies for surface lineage markers and TAMs as follows: APC-CD11c (BD 559877), PerCP-CD14 (BD 340585), PE anti-Axl (R & D FAB154P), anti-Mer (FAB8912P), and anti-Tyro (FAB859P). The immunostained cells were washed with BD wash buffer and fixed in 1% paraformaldehyde. Data were acquired using an LSR II instrument (BD Biosciences, CA) and analyzed using FlowJo software.

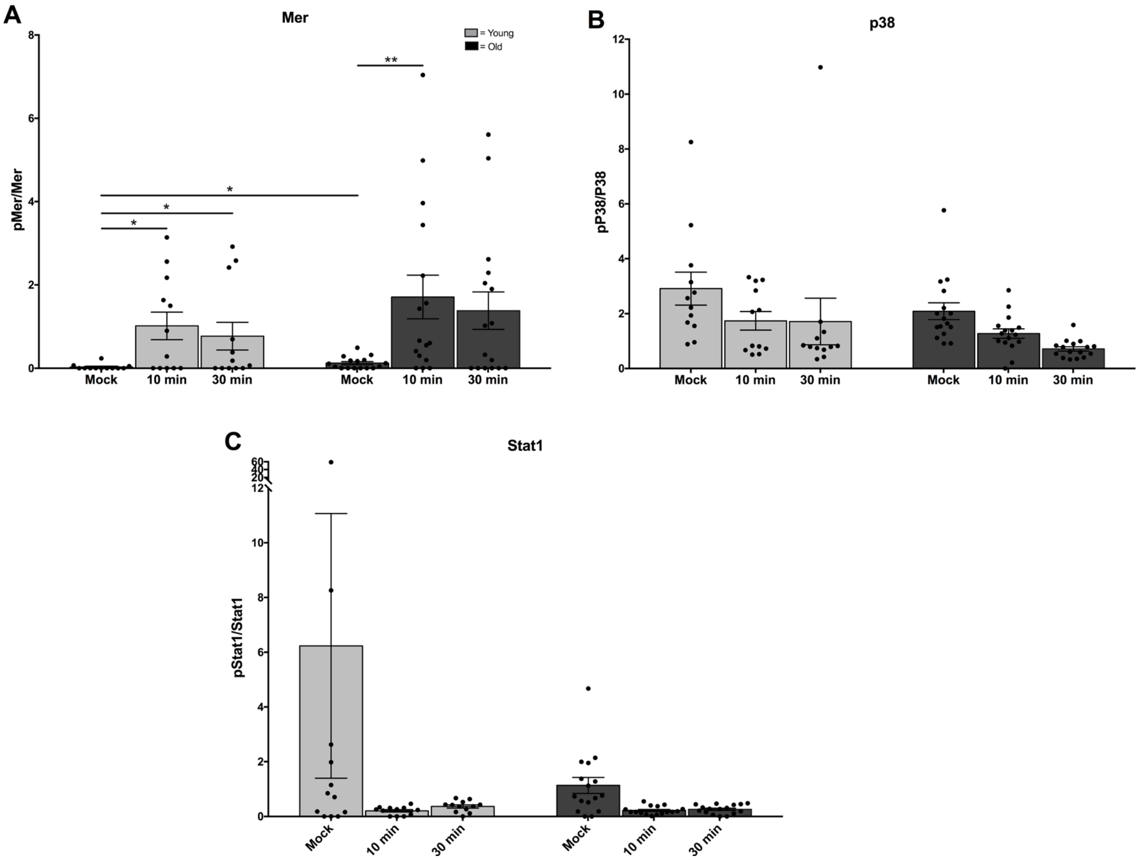

Supplementary Figure 2: Monocytes from younger and older adults were incubated overnight in the absence of serum before treatment with 0.5mM phosphatidylserine liposomes containing 100 nM Protein S. Cells were harvested at the time points indicated and lysates were processed for Immunoblot. Data shown is densitometry for N = 12 young, *n* = 16 old.
